# Integrated bulk and single-cell transcriptomic analyses suggest a myeloid-associated candidate role of *THBS1* in sepsis

**DOI:** 10.3389/fcimb.2026.1891550

**Published:** 2026-10-26

**Authors:** Chen Chen, Yantong Jin, Wei Chen, Hua Yuan, Yingchun Hu, Juan Hu

**Affiliations:** 1 Department of Emergency Medicine, The Affiliated Hospital of Southwest Medical University, Luzhou, Sichuan, China; 2 Department of Emergency Medicine, Leshan People’s Hospital, Leshan, Sichuan, China; 3 Department of Emergency Medicine, Suining First People’s Hospital, Suining, Sichuan, China; 4 Skin Structure and Function Key Laboratory of Luzhou, Department of Dermatology, Luzhou, Sichuan, China; 5 Department of Respiratory and Critical Care Medicine, The Affiliated Hospital of Southwest Medical University, Luzhou, Sichuan, China

**Keywords:** molecular docking, myeloid cells, prognosis, sepsis, single-cell RNA sequencing, *THBS1*, transcriptomics

## Abstract

**Background:**

Sepsis remains a major cause of mortality worldwide, and biomarkers that integrate inflammatory dysregulation, immune-cell localization, and prognostic relevance remain limited. To identify a candidate molecule linked to myeloid immune responses in sepsis, we performed an integrated analysis of public bulk transcriptomic datasets, single-cell RNA sequencing data, a small exploratory clinical RNA-sequencing cohort, and compound screening analyses.

**Results:**

Differential expression analysis and meta-analysis across public datasets identified *THBS1* as a consistently altered transcript in sepsis. In a small exploratory peripheral-blood transcriptomic cohort comprising 23 patients with sepsis and 10 healthy controls, *THBS1* was selected for downstream analysis on the basis of its position in the public-data protein–protein interaction network and the subsequent *THBS1*-focused analyses. The reused clinical RNA-sequencing cohort was retained only as an exploratory description and was not used to claim independent validation. Single-cell analysis suggested that *THBS1* expression was more prominent in subsets of myeloid-related cell populations. Survival analysis in an external dataset indicated that high *THBS1* expression was associated with poorer outcomes, but this finding should be interpreted as exploratory because formal adjustment for disease severity was not possible. Candidate compounds associated with *THBS1* were further prioritized by database mining and molecular docking.

**Conclusions:**

This study provides transcriptomic and computational evidence suggesting that *THBS1* may be associated with myeloid-related immune activity and adverse prognosis in sepsis. Because the clinical cohort was small, protein-level validation and functional experiments were not performed, and substantial heterogeneity was observed across public datasets, these findings should be interpreted as preliminary and hypothesis-generating.

## Introduction

Sepsis is a life-threatening syndrome caused by a dysregulated host response to infection and is characterized by systemic inflammation, immune dysfunction, and multiple-organ failure (Singer et al., 2016; van der Poll et al., 2017; Cao et al., 2023). Its pathogenesis is complex and involves inflammatory-cell activation, immune paralysis, metabolic dysregulation, endothelial injury, coagulopathy, and regulated cell death (van der Poll et al., 2017; Cao et al., 2023). Global epidemiological studies indicate that sepsis imposes a substantial disease burden, with marked regional and socioeconomic disparities (Fleischmann et al., 2016; Rudd et al., 2020). Therefore, identifying clinically relevant molecular markers remains important for improving early risk stratification, prognostic assessment, and the development of more precise therapeutic strategies in sepsis.

Recent advances in molecular biology, immunology, and multi-omics technologies have shifted sepsis biomarker research from conventional inflammatory indices toward host-response signatures and immune-regulatory molecules (Sweeney et al., 2015; Davenport et al., 2016; He et al., 2024; Li et al., 2025). Although multi-marker models may improve diagnostic and prognostic performance, clinically interpretable targets that connect inflammatory dysregulation, immune-cell localization, prognosis, and potential therapeutic relevance remain limited. Differentially expressed genes may contribute to the early identification and prognostic stratification of patients with sepsis, whereas computational compound screening may provide preliminary molecular leads for future experimental validation.

A previous study from our group used GSE134347, GSE167363, GSE220189, and the institutional RNA-sequencing cohort CNP0002611 to investigate a sepsis biomarker panel centered on CD27, KLRB1, RETN, and CD163 (Li et al., 2025). The present study reuses these datasets and several upstream analytical steps to address a narrower question focused on *THBS1*. Its additional analyses include an exploratory *THBS1*-specific survival assessment, a cross-cohort synthesis of *THBS1* expression, HERB 2.0-based compound prioritization, and molecular docking. These additions do not constitute independent validation of the reused cohorts or demonstrate therapeutic efficacy. The shared data, reanalyses, and new *THBS1*-specific components are compared in **Supplementary Table S1**. In this study, we integrated public bulk microarray data, single-cell RNA-sequencing data, and a previously reported exploratory peripheral-blood RNA-sequencing cohort to investigate *THBS1* in sepsis. Differential-expression, enrichment, and protein–protein interaction analyses were used to prioritize *THBS1*. We then examined its transcript-level association across cohorts, explored survival in a public dataset, and described its distribution across immune-cell populations (Fang et al., 2021; Gao et al., 2025). Finally, HERB 2.0 was used to prioritize candidate compounds for exploratory molecular docking. These computational analyses generate hypotheses for subsequent experimental testing (**Figure 1**).

**Figure 1:**
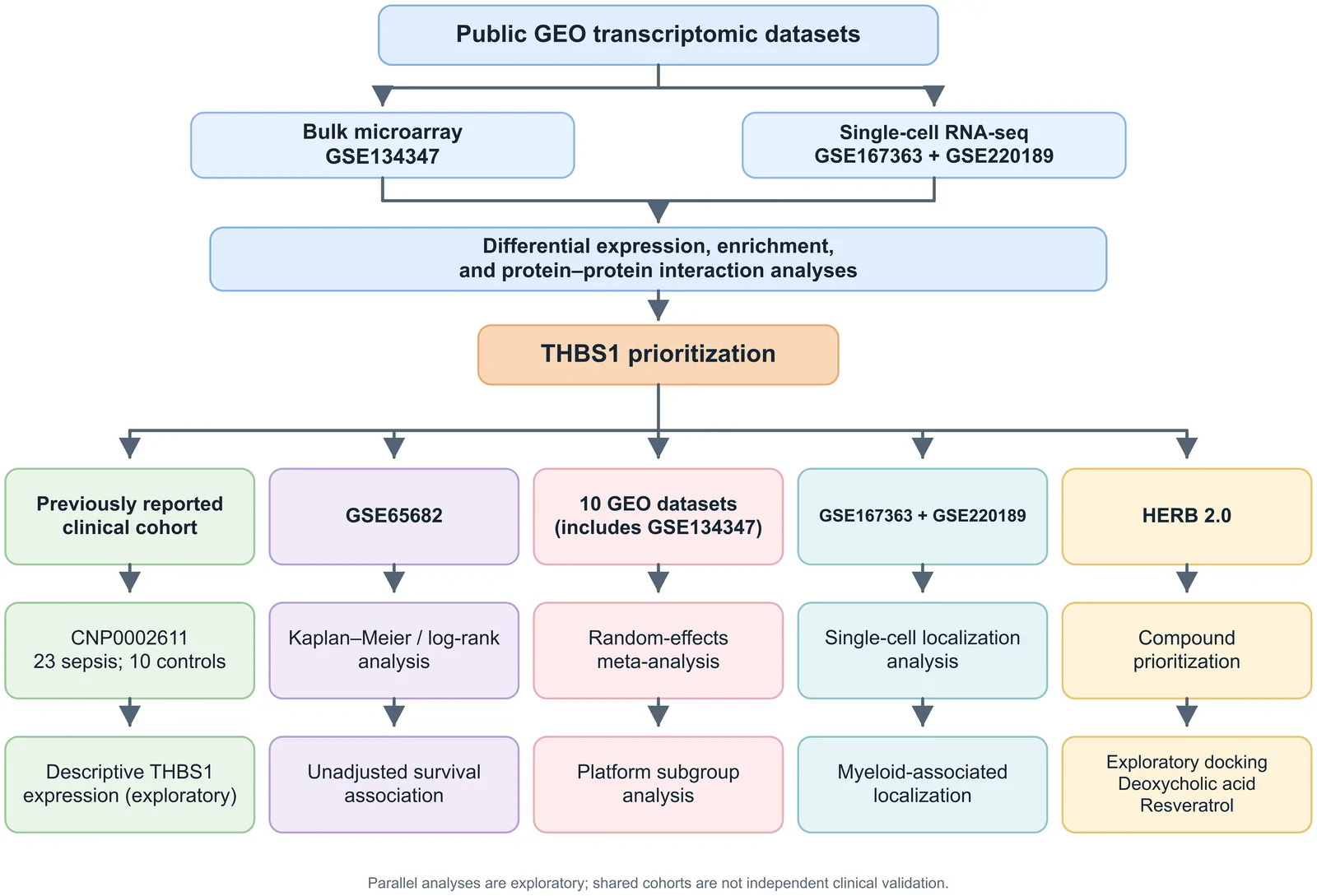
Study workflow. Public bulk and single-cell transcriptomic datasets were analyzed to prioritize *THBS1*, followed by exploratory collected for RNA sequencing clinical cohort, survival analysis, cross-cohort expression synthesis, and immune-cell localization. HERB 2.0 was used to prioritize compounds for exploratory molecular docking. All compound-related results are computational and require experimental validation.

## Materials and methods

### Data acquisition and study design

This study included three sepsis-related public datasets from the Gene Expression Omnibus (GEO) database for the identification and validation of differentially expressed mRNAs in sepsis, comprising one bulk microarray dataset (GSE134347) and two single-cell RNA sequencing datasets (GSE167363 and GSE220189). GSE134347 was generated using the Affymetrix Human Transcriptome Array 2.0 platform and contains transcriptomic profiles from 156 patients with sepsis and 83 healthy controls. To balance sample sizes between groups and reduce analytical heterogeneity, 50 sepsis samples and 50 healthy control samples were randomly selected from GSE134347 for bulk transcriptomic analysis. Principal component analysis (PCA) was performed for sample quality control, and outlier samples with aberrant expression profiles were excluded. The remaining data were then normalized before downstream analysis. Differentially expressed genes were identified from the normalized expression matrix using an absolute log₂ fold-change cutoff of 1 and FDR < 0.05. The bulk transcriptomic analyses were conducted using iDEP 0.96 and the Xiantao Academic online platform (Ge et al., 2018).

To distinguish this study from our previous publication (Li et al., 2025), **Supplementary Table S1** identifies the reused datasets and analyses alongside the new *THBS1*-specific components. GSE134347, GSE167363, GSE220189, and the institutional cohort CNP0002611 were used in both studies. The present work reanalyzes these sources for *THBS1* and adds an exploratory *THBS1* survival assessment, a multi-cohort expression synthesis, HERB 2.0-based compound prioritization, and molecular docking. The shared clinical samples are not presented as an independent validation cohort.

### Functional enrichment analysis

Gene Ontology and Kyoto Encyclopedia of Genes and Genomes enrichment analyses were performed to characterize the biological functions and pathways associated with differentially expressed genes (Ashburner et al., 2000; Kanehisa and Goto, 2000; Geistlinger et al., 2021). Gene Ontology analysis included biological process, cellular component, and molecular function categories. Kyoto Encyclopedia of Genes and Genomes analysis was used to identify enriched biological pathways. Enrichment analyses and visualizations were performed in R (Gentleman et al., 2005). P values were adjusted for multiple testing using the Benjamini-Hochberg method. Adjusted P < 0.05 was considered statistically significant. Enrichment results were visualized using bar plots and bubble plots.

### Protein-protein interaction network analysis

Overlapping differentially expressed genes between the bulk transcriptomic dataset and the single-cell RNA-sequencing datasets were identified using Venn analysis. Protein-protein interaction analysis of the overlapping genes was performed using the STRING database, with Homo sapiens selected as the organism (Szklarczyk et al., 2023). The minimum required interaction score was set to 0.4 to balance network coverage and confidence. The resulting interaction network was used for visualization and topological analysis. Key modules and hub genes were identified according to network connectivity and module structure (Biswas et al., 2020). Genes with high network connectivity and biological relevance to sepsis-associated inflammation were prioritized for further validation.

### Clinical cohort and RNA sequencing

This study enrolled 23 patients with sepsis and 10 healthy controls from the emergency intensive care unit of the Affiliated Hospital of Southwest Medical University. Peripheral blood samples were collected for RNA sequencing to explore the expression levels of hub targets identified from public datasets. The clinical RNA-seq data have been deposited in the China National GeneBank Database under accession number CNP0002611. This dataset was generated by our research group and was partially described in a previously published study (Li et al., 2025). In the present study, this dataset was reintegrated with additional public transcriptomic and single-cell data to explore the prognostic and therapeutic relevance of *THBS1* in sepsis. Because this clinical RNA-sequencing cohort was relatively small and derived from an existing single-center sequencing project, no formal prospective sample-size calculation was performed. Therefore, this cohort was used as an exploratory pilot cohort rather than a definitive clinical validation cohort. No formal inference regarding diagnostic accuracy, prognostic independence, or clinical utility was made from this cohort. Findings from this cohort should be interpreted as preliminary and require confirmation in larger prospective multicenter cohorts.

### Clinical cohort and sample collection

Patients with sepsis were enrolled from the emergency intensive care unit of the Affiliated Hospital of Southwest Medical University. Sepsis was diagnosed according to the Sepsis-3 criteria (Singer et al., 2016). Healthy controls were recruited from individuals undergoing routine physical examination and had no clinical evidence of acute infection, autoimmune disease, malignant tumor, chronic inflammatory disease, or recent immunosuppressive treatment. Peripheral blood samples were collected after enrollment. For patients with sepsis, blood samples were collected as early as possible after diagnosis or admission to the emergency intensive care unit. The exact sampling time was not uniformly recorded and was therefore not used for formal temporal analyses. Peripheral blood was collected into EDTA anticoagulant tubes and processed as soon as possible for RNA extraction. Total RNA was extracted using TRIzol reagent, and RNA quality was assessed using an Agilent 2100 Bioanalyzer before library preparation.

Peripheral blood samples were collected from all participants for RNA sequencing. Clinical laboratory indicators, including alanine aminotransferase, aspartate aminotransferase, direct bilirubin, total bilirubin, creatinine, urea, uric acid, white blood cell count, neutrophil count, monocyte count, and lymphocyte count, were collected from medical records. The dataset generated and analyzed in this study is available in the China National GeneBank Database (CNGBdb) repository at https://db.cngb.org/ under accession number CNP0002611. This clinical cohort was generated by our research group and was partially described in a previous study (Li et al., 2025). In the present study, this dataset was reintegrated with additional public transcriptomic and single-cell datasets to evaluate the prognostic and therapeutic relevance of *THBS1* in sepsis. SOFA scores, APACHE II scores, procalcitonin, C-reactive protein, lactate, and IL-6 were not uniformly available for all patients; therefore, stratified severity analyses, multivariable prognostic adjustment, and direct comparisons with conventional biomarkers were not performed.

All participants or their legally authorized representatives provided written informed consent. This study was approved by the Ethics Committee of the Affiliated Hospital of Southwest Medical University, Luzhou, China, under approval number ky2018029, and was registered in the China National GeneBank Database (CNGBdb) repository at https://db.cngb.org/ under accession number CNP0002611.

### RNA sequencing of clinical samples

Total RNA was extracted from peripheral blood samples using TRIzol reagent. RNA concentration and quality were assessed using an Agilent 2100 Bioanalyzer. Ribosomal RNA was removed using an RNase H-mediated depletion method after hybridization with specific oligonucleotides. RNA samples were fragmented at high temperature in the presence of divalent cations and purified using SPRI magnetic beads. First-strand cDNA was synthesized using reverse transcriptase and random primers, followed by second-strand cDNA synthesis using DNA polymerase I and RNase H.

Library fragment-size distribution was assessed using an Agilent 2100 Bioanalyzer, and libraries were quantified by TaqMan probe-based quantitative PCR. Paired-end sequencing was performed on the BGISEQ-500/MGISEQ-2000 platform at BGI-Shenzhen. Raw sequencing reads were filtered using SOAPnuke to remove adaptor sequences, reads with high proportions of unknown bases, and low-quality reads. Clean reads were saved in FASTQ format. After normalization of gene-expression data, the expression levels of candidate hub genes were compared between patients with sepsis and healthy controls. Normalized expression values were used for visualization. Between-group differences were assessed using the independent-samples t test when data were normally distributed; otherwise, the Mann-Whitney U test was used. P < 0.05 was considered statistically significant.

### Exploratory assessment of hub gene expression

Peripheral blood RNA-seq data from the clinical cohort were used to explore the expression levels of candidate hub genes identified from the public datasets and protein-protein interaction network analysis. After normalization of raw sequencing data, expression differences between patients with sepsis and healthy controls were evaluated. Boxplots were generated using the Xiantao Academic online analysis platform and R. Candidate hub genes were prioritized on the basis of the public-data protein–protein interaction network; the reused clinical cohort was used only for exploratory expression assessment.

### Survival analysis

The prognostic relevance of candidate hub genes was evaluated using the public sepsis dataset GSE65682, which contains peripheral blood transcriptomic data and clinical follow-up information from patients with sepsis (Scicluna et al., 2017; Zhang et al., 2020; Yin et al., 2022). Patients were divided into high- and low-expression groups according to the median expression level of each candidate gene. Kaplan-Meier survival curves were generated in R using the survival and survminer packages. Differences between groups were evaluated using the log-rank test. A two-sided P value < 0.05 was considered statistically significant. However, because SOFA and APACHE II scores were not uniformly available, a combined prognostic model incorporating *THBS1* and established severity scores could not be constructed.

### Meta-analysis of *THBS1* expression

To evaluate the robustness of differential *THBS1* expression across independent cohorts, a meta-analysis was performed using public GEO datasets, including GSE163151, GSE232753, GSE95233, GSE69063, GSE243217, GSE236713, GSE185263, GSE154918, GSE134347, and GSE100159. The total sample size included 867 patients with sepsis and 307 healthy controls. Standardized mean difference was used as the pooled effect size because expression values were derived from different platforms and preprocessing pipelines (Toro-Dominguez et al., 2021).

Heterogeneity across studies was assessed using Cochran’s Q test and the I² statistic. Because substantial heterogeneity was observed, a random-effects model was used for pooled analysis. P < 0.05 was considered statistically significant. For each GEO dataset, the study-specific standardized mean difference (SMD), 95% confidence interval (CI), and random-effects weight are provided in **Supplementary Table S3**. Leave-one-out sensitivity results are provided in **Supplementary Table S4**. In an additional exploratory analysis, datasets were stratified by assay type (microarray or RNA sequencing) according to their GEO platform records. Study-level SMDs were pooled within each platform group using a DerSimonian–Laird random-effects model, with standard errors reconstructed from the reported 95% CI widths; the subgroup-specific SMD, 95% CI, and I² were reported in **Supplementary Table S8**. A valid quantitative analysis by anatomical infection source was not possible because source-specific *THBS1* effect estimates were unavailable for the mixed-source cohorts.

Leave-one-out sensitivity analysis was performed using the R package metafor. The same study-level standardized mean differences and standard errors used in the primary meta-analysis were entered into a random-effects model. Each dataset was sequentially omitted to evaluate whether the pooled effect estimate was disproportionately influenced by any individual dataset.

### Single-cell localization of *THBS1*

A total of 12 single-cell samples were analyzed. Raw reads were processed using Cell Ranger (v8.0.1) for quality control and gene quantification (Zheng et al., 2017). Following the initial Cell Ranger filtering, further quality control was performed in Seurat (v4.0.0). Cells were retained if they met the following criteria: nFeature_RNA ≥ 200, nCount_RNA ≥ 1000, log10GenesPerUMI ≥ 0.7, mitochondrial gene proportion ≤ 30%, and hemoglobin gene proportion ≤ 5%. Putative doublets were removed using DoubletFinder (v2.0.3). The number of cells retained after quality control and after doublet removal was recorded for each sample and is provided in **Supplementary Table S4**. After quality control and doublet removal, the data were normalized using the Seurat NormalizeData function.

The top 2000 highly variable genes were selected for dimensionality reduction. Principal component analysis (PCA) was performed, and UMAP was used for visualization. When batch effects were present, Harmony was applied for batch correction. Cell clustering was performed using a graph-based clustering approach in Seurat. Cell identities were annotated using SingleR (v1.4.1) with the Human Primary Cell Atlas (HPCA) reference dataset and canonical marker genes (Aran et al., 2019).

The expression distribution of *THBS1* was visualized across major peripheral blood immune-cell subsets, including T cells, monocytes/macrophages, B cells, natural killer cells, and neutrophils. Cell-type-specific expression patterns were used descriptively to visualize the immune-cell populations associated with *THBS1* expression in sepsis.

### Candidate compound screening

Candidate compounds associated with *THBS1* were screened using HERB 2.0 (Fang et al., 2021; Gao et al., 2025). Database-reported treatment-associated changes in *THBS1* expression were examined. Deoxycholic acid and resveratrol were selected for exploratory molecular docking based on drug-likeness, as detailed in the **Supplementary Material**.

### Molecular docking

The three-dimensional structure of *THBS1* used as the docking receptor was the human *THBS1* N-terminal domain crystal structure (PDB ID: 2ERF; resolution: 1.45 Å; Homo sapiens; chain A; residues 7–215, corresponding to UniProt residues 25–233). This structure was selected because it is a high-resolution X-ray crystal structure of *THBS1* without a co-crystallized ligand. The receptor was prepared using PyMOL 2.6 by retaining chain A and removing crystallographic water molecules and other non-protein heteroatoms. The cleaned receptor was then saved in PDB format.

Ligand and receptor preparation was performed using AutoDockTools 1.5.6. Polar hydrogens were added to the receptor, and Gasteiger charges were assigned. Hydrogen atoms were added to ligand molecules, rotatable bonds were defined, and ligand structures were converted to PDBQT format. The docking grid box was defined to cover the predicted binding region of *THBS1*. Molecular docking was performed using AutoDock Vina (Trott and Olson, 2010; Eberhardt et al., 2021). Docking conformations were ranked according to predicted binding energy, and the conformation with the lowest binding energy was selected as the optimal binding pose (Sahoo et al., 2022).

Binding energies were reported in kcal/mol. A lower binding energy indicates stronger predicted binding affinity and a more stable ligand-receptor conformation. In this study, binding energy < -5.0 kcal/mol was considered indicative of favorable predicted binding activity. Docking results were visualized using Discovery Studio 2019 and PyMOL 2.6 to generate two-dimensional and three-dimensional interaction diagrams showing hydrogen bonds, hydrophobic interactions, and interactions with key amino-acid residues.

### Statistical analysis

Differential-expression analysis for the bulk microarray dataset was performed using a microarray-compatible workflow, and multiple testing correction was performed using the Benjamini-Hochberg method. Survival differences were assessed using Kaplan-Meier analysis and the log-rank test. Meta-analysis was performed using standardized mean difference as the effect size, and a random-effects model was applied when substantial heterogeneity was present. Leave-one-out sensitivity analysis was performed using the R package metafor. Unless otherwise stated, P < 0.05 was considered statistically significant.

### Bulk transcriptomic analysis

Bulk transcriptomic analysis was performed using GSE134347. Fifty sepsis samples and 50 healthy controls were randomly selected for comparison. PCA was used for quality control to identify aberrant samples. Differentially expressed genes were identified using iDEP 0.96 and the Xiantao Academic platform, with an absolute log₂ fold-change cutoff of 1 and FDR < 0.05.

### Single-cell transcriptomic analysis

Single-cell data from GSE167363 and GSE220189 were integrated for downstream analysis. Five sepsis patients and two healthy individuals from GSE167363, together with five healthy individuals from GSE220189, were included. Cells were filtered using the following criteria: nFeature_RNA ≥ 200, nCount_RNA ≥ 1000, log10GenesPerUMI ≥ 0.7, mitochondrial gene percentage ≤ 30%, and hemoglobin gene percentage ≤ 5%. The retained cells were normalized, and putative doublets were removed using DoubletFinder. The top 2,000 highly variable genes were selected for downstream analysis. PCA, SNN clustering, and UMAP were used for dimensionality reduction, clustering, and visualization. Harmony was applied to reduce batch effects using sample/dataset identity as the batch covariate. Cell types were annotated using SingleR. Differentially expressed genes in the integrated single-cell analysis were defined using P < 0.05 and |log₂FC| > 0.585. Use subscripts for log₂. The cutoff follows the more precise corner labels in **Figure 2D**; please also correct that panel's title to 0.585 as separately annotated.

### Functional enrichment and PPI network analysis

GO and KEGG enrichment analyses were performed using the CNSknowall online platform based on previously identified differentially expressed mRNAs. For protein-protein interaction analysis, the STRING database was queried with Homo sapiens as the organism, and the minimum interaction score was set to 0.4. Genes overlapping across analyses were used to construct the PPI network.

### Clinical expression assessment and survival analysis

Candidate biomarkers were explored in the CNGBdb cohort CNP0002611, which included 23 sepsis patients and 10 healthy controls. *THBS1* expression in the exploratory clinical cohort was compared between groups using a two-sided exact Mann–Whitney U test. Survival analysis used the GSE65682 dataset, with Kaplan–Meier curves compared using the log-rank test.

### Immune-cell localization analysis

To localize candidate genes within immune-cell subsets, single-cell transcriptome data were further analyzed after Harmony correction and UMAP visualization. The presto algorithm was used to identify cell-type-associated genes with logFC > 0 and min.pct > 0.25. SingleR was used to assist in cell-type annotation. This analysis was used to localize *THBS1* expression across immune-cell subsets.

## Results

### Identification of differentially expressed mRNAs in sepsis

In the GSE134347 dataset, standardization and quality control analysis showed a uniform distribution and strong intra-group consistency, highlighting significant transcriptomic differences between the sepsis and healthy control groups (**Figures 2A, B**). Differential-expression analysis identified 718 differentially expressed mRNAs in patients with sepsis compared with healthy controls, including 299 upregulated and 419 downregulated genes (**Figure 2C**). Integrated single-cell RNA-sequencing analysis of GSE167363 and GSE220189 identified 243 differentially expressed mRNAs, including 153 upregulated and 90 downregulated genes (**Figure 2D**). These results indicated substantial transcriptomic alterations in patients with sepsis at both the bulk transcriptomic and single-cell levels.

**Figure 2:**
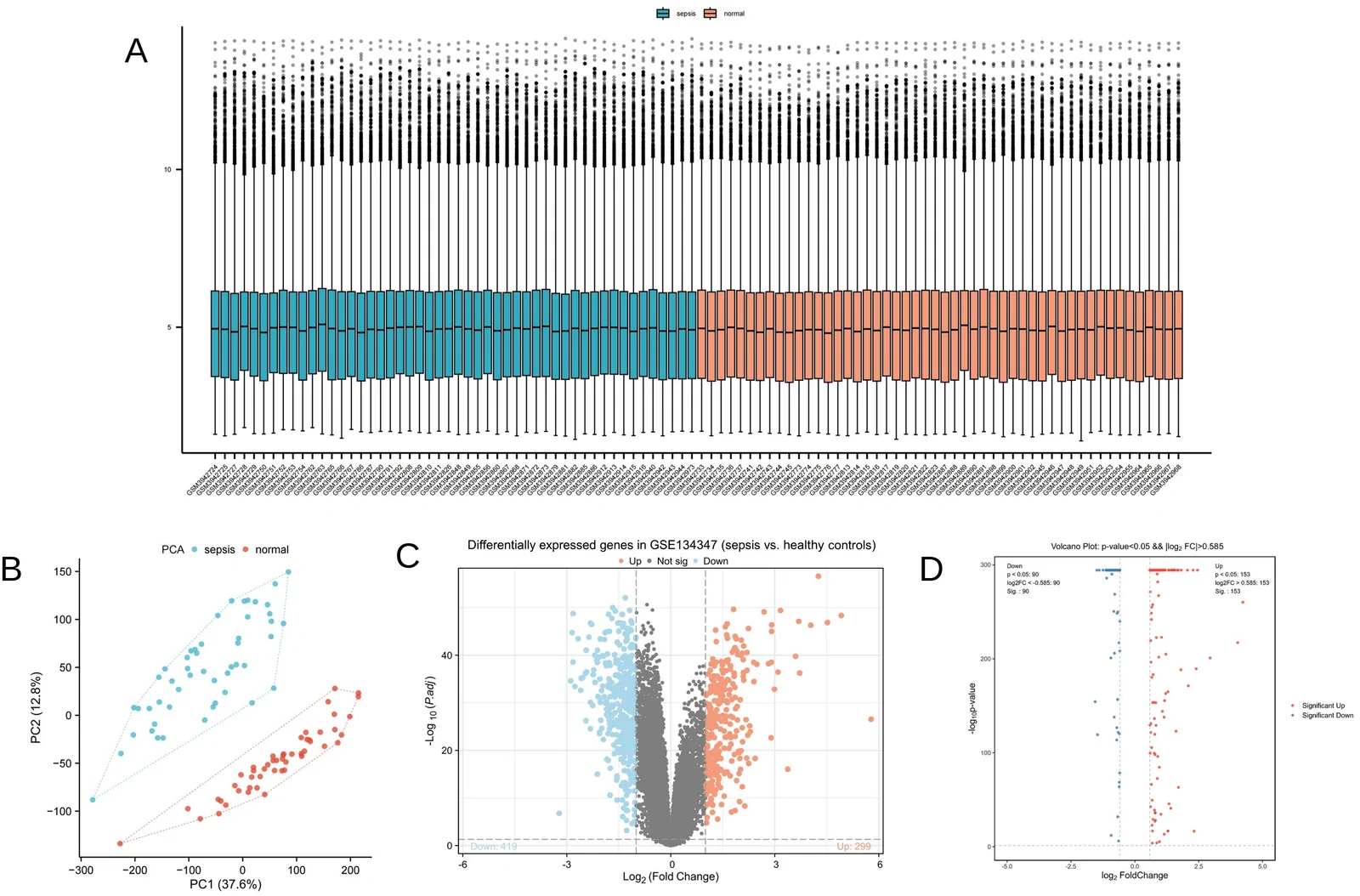
Data quality control and identification of differentially expressed genes. **(A)** Boxplot showing the expression distribution of samples in the GSE134347 dataset after standardization and quality control. **(B)** Principal component analysis showing separation between patients with sepsis and healthy controls. **(C)** Volcano plot showing differentially expressed genes in the GSE134347 dataset. **(D)** Volcano plot showing differentially expressed genes identified from the integrated single-cell RNA-sequencing analysis. Red indicates significantly upregulated genes, blue indicates significantly downregulated genes, and grey indicates genes without significant differential expression.

### Functional enrichment of inflammation- and immune-related pathways

Gene Ontology enrichment analysis of differentially expressed genes from the GSE134347 dataset showed that these genes were mainly enriched in immune-related cellular components and molecular functions. Kyoto Encyclopedia of Genes and Genomes pathway analysis further showed enrichment in inflammation- and immunity-related pathways (**Figure 3A**).

**Figure 3:**
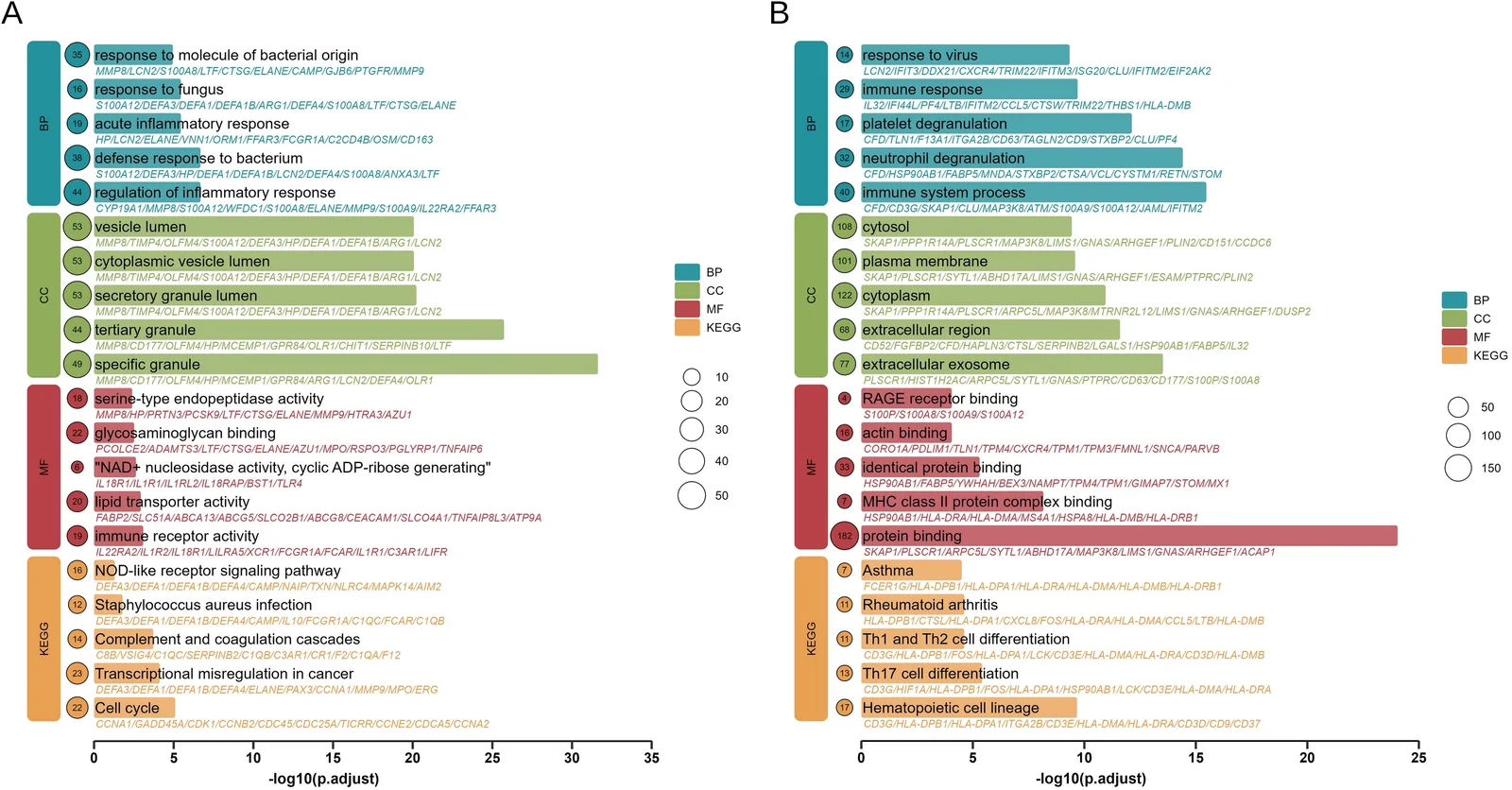
GO and KEGG enrichment analysis of differentially expressed genes. Gene Ontology and Kyoto Encyclopedia of Genes and Genomes enrichment analyses were performed for differentially expressed genes identified from the bulk transcriptomic and single-cell RNA-sequencing datasets. **(A)** Enrichment results for differentially expressed genes from the GSE134347 dataset. **(B)** Enrichment results for differentially expressed genes from the integrated single-cell RNA-sequencing datasets. Thex-axis represents −log_10_(adjusted P value), and the y-axis represents enriched terms.

In the single-cell RNA-sequencing datasets, Gene Ontology enrichment analysis showed that differentially expressed genes were mainly involved in antiviral responses, immune responses, and immune system processes. Kyoto Encyclopedia of Genes and Genomes analysis indicated enrichment in immune-related pathways (**Figure 3B**).

### Protein-protein interaction network analysis identified *THBS1* as a hub target

Venn analysis identified 57 overlapping differentially expressed mRNAs between the GSE134347 bulk microarray dataset and the single-cell RNA-sequencing dataset (**Figure 4A**). Protein-protein interaction network analysis of these overlapping genes revealed two major hub modules (**Figure 4B**). One module was centered on CD3D, CD2, GZMK, and CCL5, whereas the other module was centered on *THBS1*, MMP9, and PPBP. Clinical RNA-seq data were examined descriptively in the reused exploratory cohort; the *THBS1* results are presented below. Given its high network connectivity, differential expression in public datasets, and potential prognostic relevance, *THBS1* was selected as the key candidate target for subsequent analyses.

**Figure 4:**
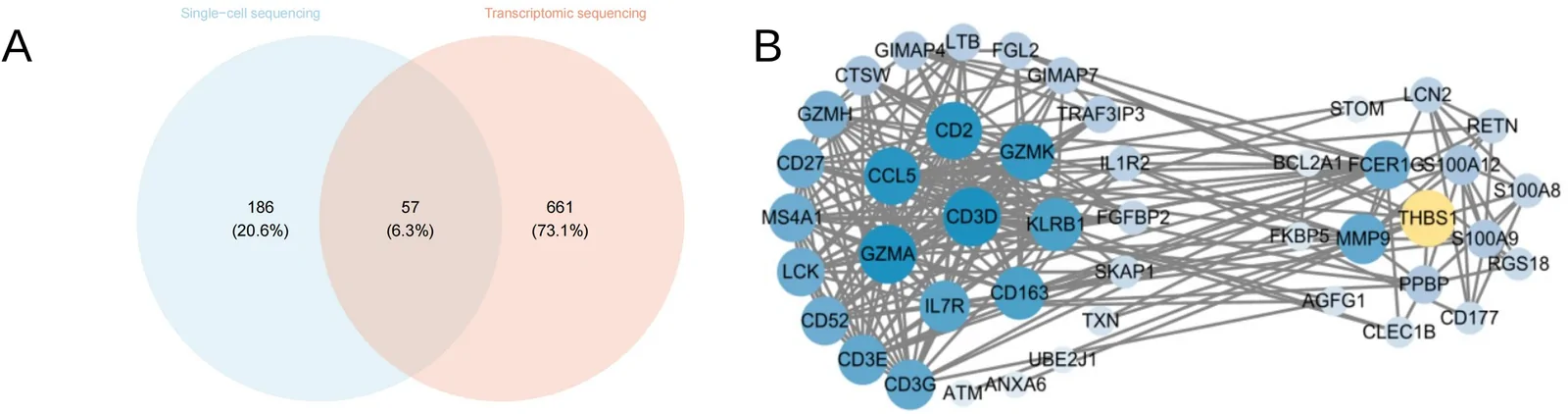
Screening of hub targets. **(A)** Venn diagram showing overlapping differentially expressed genes between the GSE134347 dataset and the single-cell RNA-sequencing datasets. **(B)** Protein-protein interaction network showing two major hub modules.

### Clinical characteristics of the exploratory cohort

The clinical characteristics of the 23 patients with sepsis and 10 healthy controls are summarized in **Supplementary Table S1**. In the reused exploratory cohort (23 patients with sepsis and 10 healthy controls), the tabulated *THBS1* expression values had means of 12.510 and 10.881, respectively. The descriptive log_2_ ratio of these arithmetic means was +0.201, and the standardized mean difference (Hedges’ g) was +0.171. An exploratory two-sided exact Mann–Whitney test did not detect a between-group difference (P = 0.893). Because this cohort was small and the distribution was variable, these results do not support clinical validation. Cohort characteristics and sample-level expression are provided in **Supplementary Table S9** and **Supplementary Figure S1**.

### Prognostic relevance of *THBS1* in sepsis

Survival analysis was performed using the public sepsis dataset GSE65682. Patients were divided into high- and low-expression groups according to the median expression level of each candidate gene. Kaplan-Meier analysis showed distinct prognostic patterns among hub genes.

High expression of CD3D and CD2 was associated with better survival in patients with sepsis (**Figures 5A, B**). In contrast, high *THBS1* expression was associated with poorer survival (**Figure 5C**), whereas the association for PPBP was not statistically significant (P = 0.36; **Figure 5D**). Notably, patients with high *THBS1* expression had significantly shorter survival than those with low *THBS1* expression, with a log-rank P value of 0.0029 (**Figure 5C**). These findings suggest that high *THBS1* expression may be associated with adverse prognosis in patients with sepsis, although this association should be interpreted as exploratory because formal adjustment for disease severity was not possible.

**Figure 5:**
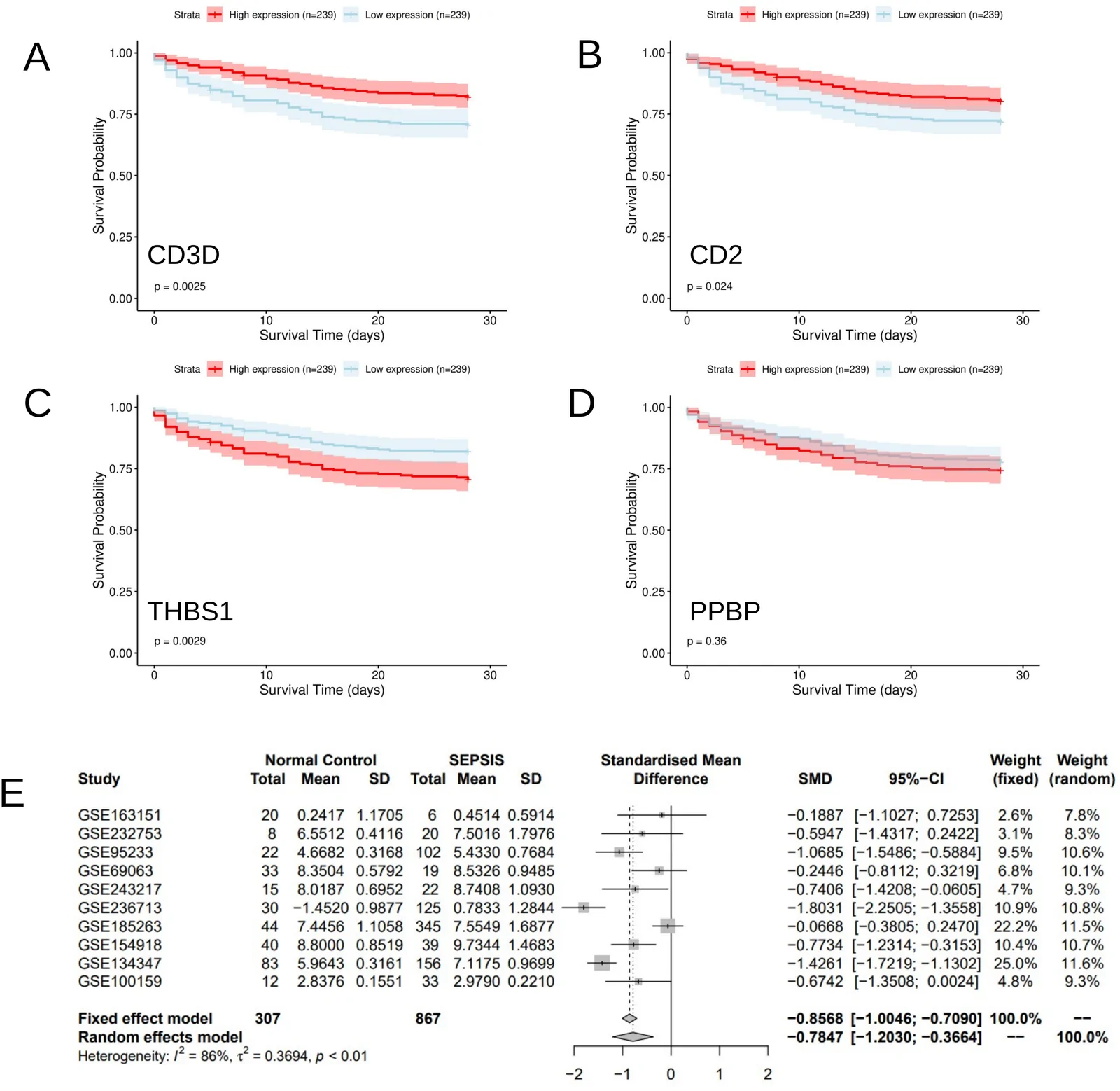
Survival analysis and meta-analysis. **(A–D)** Kaplan-Meier survival curves based on the public GSE65682 dataset. Patients were divided into high- and low-expression groups according to the median expression level of each gene. Red curves represent the high-expression group, and blue curves represent the low-expression group. High expression of CD3D and CD2 was associated with better survival, whereas high expression of *THBS1* was associated with poorer survival in exploratory unadjusted analyses. **(E)** Forest plot showing study-specific and pooled standardized mean differences in *THBS1* expression across ten GEO datasets, including GSE134347. Negative values indicate higher expression in sepsis because the contrast was defined as healthy control minus sepsis. Study-specific SMDs, 95% CIs, and random-effects weights are provided in **Supplementary Table S3**; leave-one-out results are provided in **Supplementary Table S4**. Exploratory platform-stratified results are provided in **Supplementary Table S8**.

### Meta-analysis of *THBS1* expression in public datasets

To evaluate whether differential *THBS1* expression was robust across independent cohorts, a meta-analysis was performed using ten public GEO datasets. Ten GEO datasets, including GSE163151, GSE232753, GSE95233, GSE69063, GSE243217, GSE236713, GSE185263, GSE154918, GSE134347, and GSE100159, were included in the meta-analysis. The study-specific SMDs and 95% CIs are presented in **Supplementary Table S3** and **Figure 5E**. The pooled random-effects SMD across the ten datasets, including the discovery dataset GSE134347, was −0.7847 (95% CI, −1.2030 to −0.3664). Heterogeneity was substantial (I² = 86%; τ² = 0.3694; P < 0.01). Negative SMDs denote higher *THBS1* transcript levels in sepsis because the effect was calculated as healthy control minus sepsis.

A leave-one-out sensitivity analysis was then performed using the same study-level effect estimates and standard errors. After sequential exclusion of each dataset, the pooled SMD remained negative and statistically significant, with estimates ranging from -0.897 to -0.674. The corresponding 95% CIs remained below zero. Substantial heterogeneity persisted across the leave-one-out analyses, with I² values ranging from 74.1% to 84.3%. These findings suggested that the direction and statistical significance of the pooled association were not driven by any single dataset. Detailed leave-one-out results are provided in **Supplementary Table S4**.

In the exploratory platform-stratified analysis, the pooled SMD was −1.0804 (95% CI, −1.5780 to −0.5829; I² = 82.33%) across five microarray datasets and −0.4475 (95% CI, −0.8149 to −0.0801; I² = 50.76%) across five RNA-sequencing datasets (**Supplementary Table S8**). Both pooled estimates were negative. The nominal between-platform P value was 0.0449, but this comparison was not robust to exclusion of the discovery dataset, and substantial heterogeneity remained within the microarray group. When the discovery dataset GSE134347 was excluded, the microarray subgroup estimate was −0.9680 (95% CI, −1.6520 to −0.2840; I² = 84.83%; four datasets), whereas the RNA-sequencing estimate remained −0.4475; the between-group P value was 0.1888. These findings do not establish an assay-independent effect of fixed magnitude.

The pooled analysis showed that *THBS1* expression differed significantly between patients with sepsis and healthy controls, with a moderate-to-large pooled effect size (**Figure 5E**). However, the magnitude of the study-specific effects varied across datasets, and substantial heterogeneity was observed. The ten study-specific SMD point estimates were all negative, whereas their magnitudes and precision varied across datasets and several 95% CIs included zero. The pooled SMD quantifies a standardized difference in transcript abundance; it is neither a fold change nor a measure of diagnostic performance. Given the substantial heterogeneity, the pooled estimate should be viewed as exploratory evidence of a transcript-level association rather than a precise effect that applies uniformly across sepsis populations. Expression differences between cases and controls and prognosis within sepsis patients address different questions and should be interpreted separately. The meta-analysis across ten public GEO datasets provided exploratory cross-cohort evidence. Therefore, the pooled SMD should not be interpreted as a precise universal effect estimate. Instead, the meta-analysis should be interpreted as exploratory evidence of cross-cohort association and general consistency of *THBS1* differential expression rather than definitive confirmation of a uniform biological effect. In addition, expression differences between patients with sepsis and healthy controls and prognostic implications of high versus low expression within patients with sepsis represent different analytical levels and should be interpreted separately.

### Single-cell localization of *THBS1* in immune cell subsets

Single-cell RNA-sequencing quality control was performed before clustering and cell-type annotation. A total of 12 samples were analyzed. Before additional Seurat-based quality control, 2,716 to 9,485 cells were detected per sample. After filtering according to predefined quality-control thresholds, 2,249 to 9,430 cells per sample were retained. After doublet removal using DoubletFinder, 2,197 to 8,676 cells per sample were retained for downstream analysis, with a total of 60,480 cells included. The retained cells had mean detected genes ranging from 1,173 to 1,909 per cell, mean UMI counts ranging from 4,194 to 7,859 per cell, and mean mitochondrial gene proportions ranging from 0.060 to 0.110. Detailed per-sample quality-control metrics are provided in **Supplementary Table S4**. After dimensionality reduction and clustering, the cells were annotated into populations including T cells, monocytes, B cells, NK cells, neutrophils, macrophages, dendritic cells, and progenitor-related populations (**Figure 6A**).

**Figure 6:**
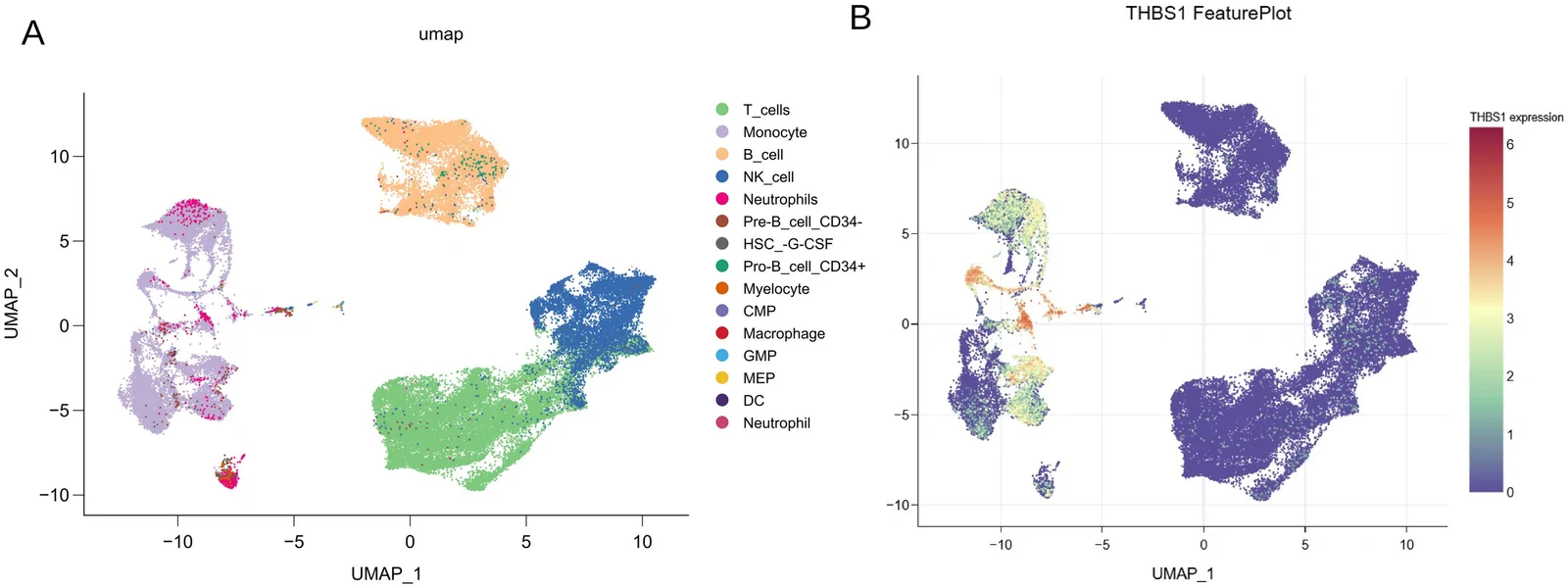
Single-cell localization of *THBS1*. **(A)** A total of 12 samples were analyzed. Before additional Seurat-based quality control, 2,716 to 9,485 cells were detected per sample, and 2,197 to 8,676 cells per sample were retained after quality control and doublet removal. Detailed per-sample quality-control metrics are provided in **Supplementary Table S4**. Cell populations were identified and annotated as shown in panel **(A)**. **(B)** Distribution of *THBS1* expression across peripheral blood immune-cell subsets. This plot is presented for visualization only, and no formal statistical testing was performed between cell types or between disease states within each cell type.

The distribution of *THBS1* expression across immune-cell subsets showed that *THBS1* was detectable across multiple sepsis-related immune-cell populations. Visually, *THBS1* appeared to be more abundant in subsets of the myeloid-related cell populations, with lower expression observed in B cells, natural killer cells, and neutrophils (**Figure 6B**). These observations are descriptive only and were not subjected to formal statistical testing.

### Screening of *THBS1*-related candidate compounds

The database-derived changes in *THBS1* expression following different treatments are shown in **Figure 7**. Both decreased and increased expression were observed across the displayed treatment records.

**Figure 7:**
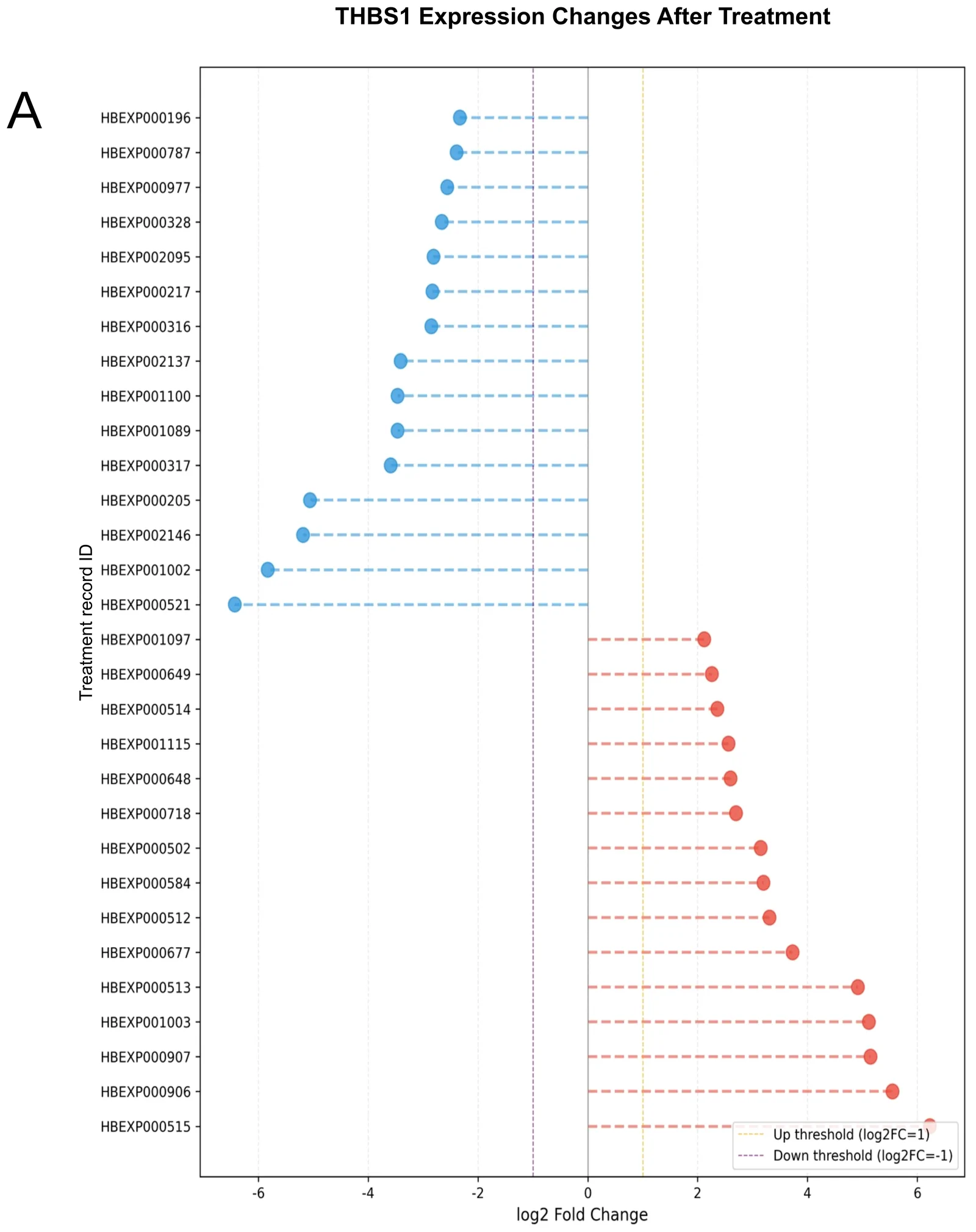
Computational screening of *THBS1*-associated candidate compounds. HERB 2.0-based computational screening of compounds associated with *THBS1*. The lollipop plot shows database-reported associations between candidate compounds and *THBS1* expression. These associations do not establish direct target engagement or anti-inflammatory activity in sepsis.

### Molecular docking of candidate compounds with *THBS1*

The HERB 2.0-prioritized compounds deoxycholic acid (PubChem CID 222528) and resveratrol (PubChem CID 445154) were docked to the human *THBS1* N-terminal structure (PDB ID: 2ERF). Their predicted binding energies were −6.6 and −6.4 kcal/mol, respectively (**Figures 8A, B**; **Supplementary Table S6**). These docking scores are computational estimates. Without experimental binding controls or functional assays, they cannot establish direct *THBS1* binding or therapeutic activity in sepsis.

**Figure 8:**
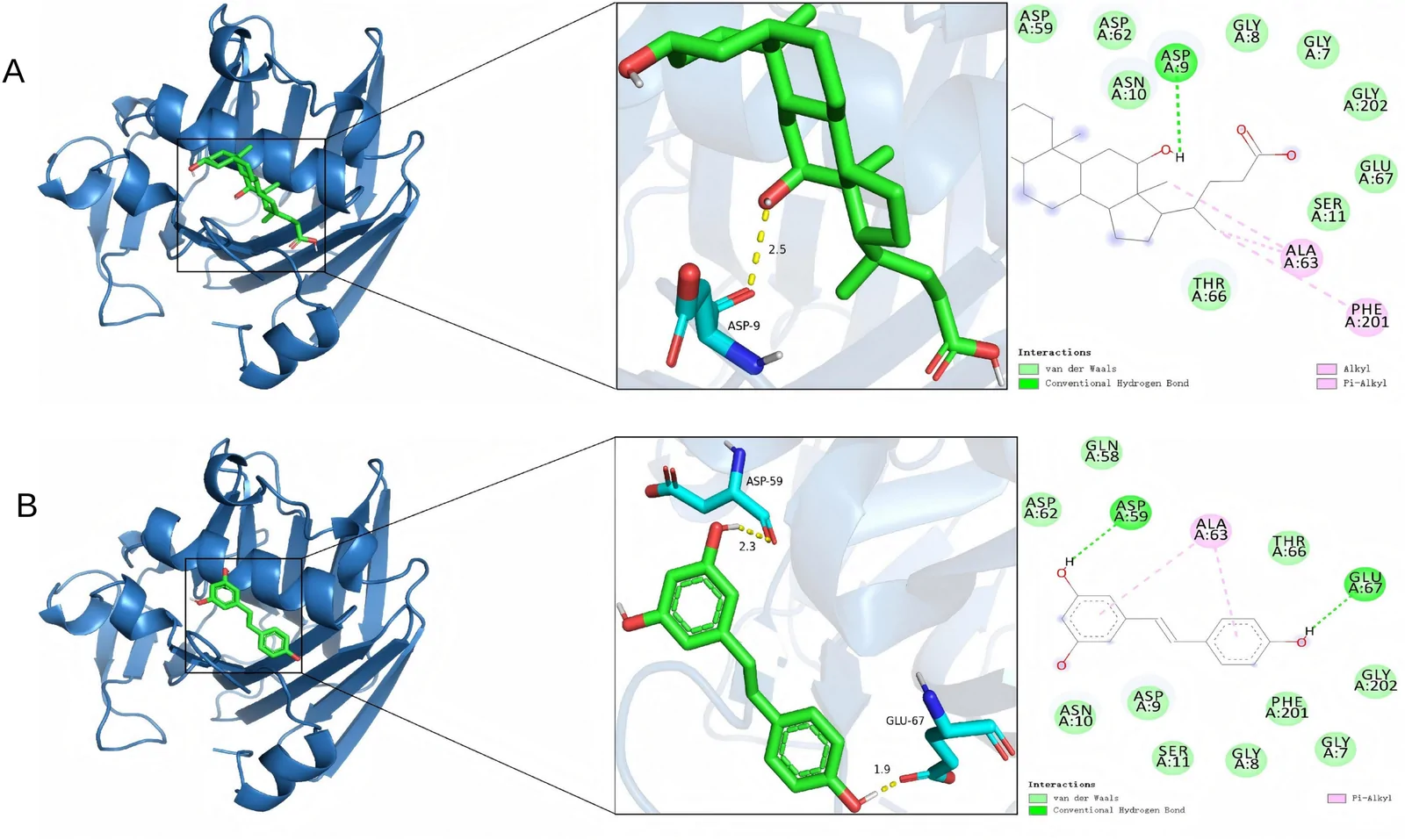
Exploratory molecular docking of two HERB 2.0-prioritized compounds with the human *THBS1* N-terminal domain (PDB ID: 2ERF; chain A). **(A)** Deoxycholic acid (PubChem CID 222528): predicted binding energy, −6.6 kcal/mol. **(B)** Resveratrol (PubChem CID 445154): predicted binding energy, −6.4 kcal/mol. Docking scores do not demonstrate experimental binding or efficacy in sepsis.

## Discussion

By integrating public bulk microarray data, single-cell RNA sequencing data, and a clinical peripheral blood RNA-sequencing cohort, this study identified *THBS1* as a candidate hub target associated with inflammatory dysregulation and adverse prognosis in sepsis. *THBS1* was prioritized through multiple layers of transcriptomic and computational evidence, including differential expression analysis, protein-protein interaction network analysis, descriptive assessment in a reused small exploratory cohort, survival analysis, cross-cohort meta-analysis, single-cell localization, compound-database screening, and molecular docking. Together, these findings suggest a potential association between *THBS1*, myeloid-related immune activity, and adverse clinical outcomes in sepsis. However, because the present study was mainly computational and transcriptome-based, these results should be interpreted as preliminary and hypothesis-generating rather than definitive mechanistic or clinical evidence.

The relationship with our previous publication (Li et al., 2025) requires a direct comparison. Both papers used the same discovery microarray dataset (GSE134347), the same two single-cell datasets (GSE167363 and GSE220189), and the same institutional RNA-sequencing cohort (CNP0002611); the 50-versus-50 discovery-sample strategy and several upstream differential-expression, enrichment, and network analyses also overlap. The earlier study identified and evaluated CD27, KLRB1, RETN, and CD163 with an emphasis on machine-learning diagnostic performance. The present paper is a reanalysis of those shared data centered on *THBS1*, supplemented by *THBS1*-specific survival analysis in GSE65682, a multi-cohort synthesis of *THBS1* expression, and exploratory compound prioritization and docking. These are new analyses of a different target, not independent replication of the shared datasets or clinical cohort. The survival association is unadjusted, the pooled expression estimate has substantial heterogeneity, and docking lacks experimental binding or efficacy controls. Consequently, our contribution is a *THBS1*-focused hypothesis and a transparent synthesis of available transcriptomic evidence, rather than proof of a new clinical biomarker or therapeutic target. *THBS1* may influence endothelial cell behavior, monocyte/macrophage-lineage activation, T-cell responses, and inflammatory signaling (Ribeiro et al., 1999; Murphy-Ullrich and Suto, 2018; Repossi et al., 2020; Suto et al., 2020; Kale et al., 2021; Kaur et al., 2021; Morandi et al., 2021; Kaur and Roberts, 2024). These previously reported biological functions provide a plausible background for the observed association between *THBS1* expression and sepsis-related immune dysregulation. Additionally, *THBS1* activates latent TGF-β through specific structural motifs, providing a potential mechanistic link to immune dysregulation in sepsis (Ribeiro et al., 1999; Murphy-Ullrich and Suto, 2018). Nevertheless, the present study did not experimentally examine the upstream regulators or downstream signaling pathways of *THBS1*; therefore, any mechanistic interpretation remains hypothetical.

The prognostic analysis showed that high *THBS1* expression was associated with poorer survival in patients with sepsis in the GSE65682 dataset. This finding is consistent with the possibility that increased *THBS1* expression may reflect a more severe inflammatory or immune-dysregulated state. However, expression differences between patients with sepsis and healthy controls and prognostic stratification based on high versus low expression within patients with sepsis represent distinct biological and analytical questions. Therefore, these results should not be interpreted as indicating that disease-control differential expression and within-disease prognostic risk are equivalent. In addition, formal disease severity scores, including SOFA and APACHE II, were unavailable or incomplete in the public survival dataset, and multivariable adjustment for baseline severity could not be performed. Thus, the prognostic association of *THBS1* should be interpreted as exploratory, and residual confounding by disease severity cannot be excluded.

Single-cell localization analysis suggested that *THBS1* was visually more abundant in subsets of myeloid-related immune-cell populations. Macrophages are central regulators of sepsis immunopathology and contribute to both early hyperinflammatory responses and later immunosuppressive states (van der Poll et al., 2017; Cao et al., 2023). In this context, *THBS1* may participate in macrophage activation, endothelial injury, inflammatory amplification, extracellular matrix remodeling, and immune suppression through latent TGF-β activation, extracellular matrix interactions, and CD47-related signaling (Ribeiro et al., 1999; Murphy-Ullrich and Suto, 2018; Kale et al., 2021). However, the single-cell analysis in this study was descriptive and visualization-based. No formal statistical testing was performed to compare *THBS1* expression between cell types or between disease states within each cell type. Therefore, direct functional validation using macrophage models, *THBS1* knockdown or overexpression, receptor-blocking experiments, and animal models of sepsis remains necessary.

The HERB 2.0 screening and docking analyses prioritized deoxycholic acid and resveratrol as exploratory *THBS1*-related candidates. Resveratrol has reported anti-inflammatory and metabolic effects (Repossi et al., 2020; Li et al., 2023; Bhuia et al., 2024), whereas deoxycholic acid is a secondary bile acid involved in metabolic and inflammatory regulation (Dayan et al., 2016; Camilleri, 2021; Evangelakos et al., 2021; Suvarna et al., 2024). However, these general biological observations do not show that either compound acts through *THBS1* in sepsis. The docking model lacked experimentally validated positive and negative controls, and favorable predicted binding energies do not establish physical binding or cellular activity. Biochemical binding assays, *THBS1* perturbation in primary immune cells, and sepsis-relevant functional models are required before any therapeutic inference can be made.

The meta-analysis across ten public GEO datasets provided exploratory cross-cohort evidence that *THBS1* expression differs between patients with sepsis and healthy controls. The observed SMD is measured in standard deviation units and should not be equated with a percentage change in *THBS1* expression, diagnostic accuracy, or biological function. Platform-stratified estimates were negative in both the microarray and RNA-sequencing groups, but the microarray subgroup retained substantial heterogeneity and the between-platform comparison was sensitive to analytic choices. Accordingly, these results support a possible transcript-level association rather than a uniform effect across technical or clinical settings. We could not estimate infection-source-specific pooled effects from the available study-level summaries because several cohorts combined patients with different anatomical infection sources. Independent cohorts with source-specific expression estimates, together with protein-level and functional studies, are needed to assess clinical and biological relevance. Leave-one-out sensitivity analysis showed that the direction and statistical significance of the pooled association were not driven by any single dataset. Nevertheless, substantial heterogeneity persisted after sensitivity analyses, indicating that differences in population characteristics, disease severity, infection source, sample type, sequencing or microarray platform, and analytical procedures may contribute to between-study variability. Therefore, the pooled standardized mean difference should not be interpreted as a precise universal effect estimate, but rather as evidence supporting the general cross-cohort association of *THBS1* with sepsis.

Several limitations should be acknowledged. First, although public datasets and a clinical RNA-sequencing cohort were integrated, the clinical cohort remained small and was derived from an existing single-center sequencing project. No formal prospective sample-size calculation was performed, and the cohort was used only as an exploratory pilot cohort. Larger prospective multicenter cohorts are needed to confirm the diagnostic and prognostic value of *THBS1* in sepsis. Second, the present study was mainly based on transcript-level analyses, and protein-level validation of *THBS1* remains lacking. Future studies should evaluate circulating and cellular *THBS1* protein levels and examine their associations with clinical severity scores, inflammatory mediators, organ dysfunction indices, and patient outcomes. Third, septic-shock status, infection-source categories, and survivor versus non-survivor subgroup comparisons could not be reliably analyzed because of the limited sample size and incomplete clinical information. Fourth, established sepsis-related biomarkers, including procalcitonin, C-reactive protein, lactate, and IL-6, were not uniformly available in the public datasets or in our exploratory RNA-sequencing cohort. Therefore, whether *THBS1* provides diagnostic or prognostic value beyond established biomarkers remains unknown. Fifth, although single-cell analysis suggested higher *THBS1* expression in subsets of myeloid-related populations, the causal role of *THBS1* in macrophage inflammatory responses, TGF-β/CD47 signaling, endothelial injury, and immune dysfunction has not been experimentally tested. Sixth, the HERB 2.0-prioritized compounds and their docking scores remain computational hypotheses because binding, *THBS1*-dependent cellular effects, and efficacy in sepsis-relevant models have not been tested. Seventh, platelet-related confounding may have influenced *THBS1* interpretation. Therefore, platelet count or activation markers should be considered in future multivariable analyses. Finally, several analyses were performed using online bioinformatics platforms; therefore, fully scripted and version-controlled computational pipelines are needed to improve reproducibility.

In summary, this study suggests that *THBS1* may be associated with myeloid-related immune dysregulation and adverse prognosis in sepsis. The findings provide a basis for future mechanistic and translational studies, but they should be considered preliminary and hypothesis-generating until validated in larger clinical cohorts and experimental models.

## Conclusion

In conclusion, this integrative transcriptomic and computational study suggests that *THBS1* may be associated with myeloid-related immune activity and adverse prognosis in sepsis. *THBS1* was supported by differential expression analysis, clinical transcriptomic assessment, survival analysis, cross-cohort meta-analysis, single-cell localization, and computational compound prioritization. However, because the clinical cohort was small and exploratory, protein-level validation and functional experiments were not performed, and substantial inter-dataset heterogeneity was observed, these findings should be considered preliminary and hypothesis-generating. Further validation in larger prospective cohorts, protein-level assays, cellular experiments, animal models, and preclinical studies is required before *THBS1* can be considered a clinically applicable biomarker or therapeutic target in sepsis.

## Data Availability

The datasets presented in this study can be found in online repositories. The names of the repository/repositories and accession number(s) can be found in the article/**Supplementary Material**.
